# Microbiological risk factors, ICU survival, and 1-year survival in hematological patients with pneumonia requiring invasive mechanical ventilation

**DOI:** 10.1007/s10096-024-04883-y

**Published:** 2024-06-26

**Authors:** Benjamin Seybold, Timo Funk, Peter Dreger, Gerlinde Egerer, Juliane Brandt, Carsten Mueller-Tidow, Nicola Giesen, Uta Merle

**Affiliations:** 1 Department of Anesthesiology, Heidelberg University Hospital, Im Neuenheimer Feld 420, 69120 Heidelberg, Germany; 2 Department of Dermatology, Venereology and Allergology, Frankfurt University Hospital, Frankfurt, Germany; 3 Department of Hematology, Oncology and Rheumatology, Heidelberg University Hospital, Heidelberg, Germany; 4 Department of Hematology, Oncology and Palliative Care, Robert Bosch Hospital, Stuttgart, Germany; 5 Department of Internal Medicine IV, Heidelberg University Hospital, Im Neuenheimer Feld 410, 69120 Heidelberg, Germany

**Keywords:** Hematological malignancies, Immunocompromised hosts, Aspergillus spp., Galactomannan, Cytomegalovirus, Invasive mechanical ventilation

## Abstract

**Purpose:**

To identify pathogenic microorganisms and microbiological risk factors causing high morbidity and mortality in immunocompromised patients requiring invasive mechanical ventilation due to pneumonia.

**Methods:**

A retrospective single-center study was performed at the intensive care unit (ICU) of the Department of Internal Medicine at Heidelberg University Hospital (Germany) including 246 consecutive patients with hematological malignancies requiring invasive mechanical ventilation due to pneumonia from 08/2004 to 07/2016. Microbiological and radiological data were collected and statistically analyzed for risk factors for ICU and 1-year mortality.

**Results:**

ICU and 1-year mortality were 63.0% (155/246) and 81.0% (196/242), respectively. Pneumonia causing pathogens were identified in 143 (58.1%) patients, multimicrobial infections were present in 51 (20.7%) patients. Fungal, bacterial and viral pathogens were detected in 89 (36.2%), 55 (22.4%) and 41 (16.7%) patients, respectively. Human herpesviruses were concomitantly reactivated in 85 (34.6%) patients. As significant microbiological risk factors for ICU mortality *probable invasive Aspergillus disease* with positive serum-Galactomannan (odds ratio 3.1 (1.2-8.0), *p* = 0.021,) and pulmonary *Cytomegalovirus* reactivation at intubation (odds ratio 5.3 (1.1–26.8), *p* = 0.043,) were identified. 1-year mortality was not significantly associated with type of infection. Of interest, 19 patients had infections with various respiratory viruses and *Aspergillus spp.* superinfections and experienced high ICU and 1-year mortality of 78.9% (15/19) and 89.5% (17/19), respectively.

**Conclusions:**

Patients with hematological malignancies requiring invasive mechanical ventilation due to pneumonia showed high ICU and 1-year mortality. Pulmonary Aspergillosis and pulmonary reactivation of *Cytomegalovirus* at intubation were significantly associated with negative outcome.

## Introduction

Hematological malignancies (HM) and cancer therapies cause diverse immune defects. Thus, these patients are at increased and multi-etiological risk of severe infectious complications. Up to 80% of patients with leukemia, lymphoma and multiple myeloma experience infectious complications during disease and treatment [1]. Development of pneumonia with acute respiratory failure (ARF) and need for invasive mechanical ventilation (MV) is associated with high mortality up to 70% [2–4].

Different hematological risk factors for mortality are known. These include hematopoietic stem cell transplantation (HSCT), graft versus host disease (gvhd) and neutropenia [5–7]. Most lower respiratory tract (LRT) infections in patients with HM are due to bacteria, often with multidrug resistance [8]. Besides community-acquired pneumonia, commonly caused by gram-positive bacteria, nosocomial infections with gram-negative germs play a crucial role in hematological patients [2].

Viral respiratory infections usually occur community-acquired and seasonally [9]. Severity ranges from common cold to severe LRT infection. In recent years the COVID-19 pandemic impressively showed the potential threat of viral pneumonia for both cancer and non-cancer patients. In patients with HM about 30% of infections with community-acquired respiratory viruses (CRV) progress to LRT infections with an associated mortality of up to 25% [8]. In case of fungal or bacterial co-infections mortality associated with viral pneumonia can be substantially higher [9].

Facultative pathogenic fungi are omnipresent, but invasive fungal infections are predominantly seen in immunocompromised patients [10]. Invasive fungal disease can be found in about 30% of patients with HM post-mortem [11]. Mostly, first site of invasive fungal infection is the lung because of the aerogenic transmission of the spores. The most common fungal pathogen detectable in LRT fluids is *Aspergillus spp.* with associated mortality rates ranging from 55 to 78% [12, 13].

Concomitant reactivation of human herpesviruses (HHV) is often found in hematological patients with pneumonia [14, 15]. Although the reported prevalence of HHV reactivation in hematological patients is high (60–70% after allogeneic HSCT) the clinical relevance is not yet fully understood [14–17]. Among HHV, especially the impact on patients’ outcome of *Cytomegalovirus (CMV)* reactivation is discussed controversially as some findings indicate a CMV reactivation-associated mortality of 57% [18, 19].

However, in 45–50% of hematological patients with clinical diagnosis of pneumonia, no pathogenic germs are found even though invasive diagnostic testing (e.g., broncho-alveolar lavage (BAL)) is performed [12, 20]. In these scenarios, radiological diagnostics, particularly computed tomography (CT) scans, are essential for verifying the site of infection and can be helpful in revealing characteristic findings indicative of specific pathogens. Even in neutropenic patients, who are usually the most challenging to diagnose due to subtle expression of clinical symptoms and radiological signs, pneumonia-specific infiltrates or even findings indicating specific pathogens have been found with a high sensitivity of up to 87% [21]. Thus, CT scans constitute a valuable complementary method to microbiological testing in identifying causative agents of pneumonia in immunocompromised patients [22].

This study was performed to help inform on prevalence of pathogens and microbiological risk factors for intensive care unit (ICU) and 1-year mortality in patients with HM and pneumonia requiring invasive MV to better predict outcome and help improve treatment strategies.

## Materials and methods

### Study design and population

This study is a retrospective single-center study. Data were collected from the ICU ward of the department of internal medicine and infectious diseases at Heidelberg University Hospital (Germany). The ICU consists of 14 treatment units equipped for intensive care including invasive MV. Annually more than 2000 patients are treated at this ICU, amongst others patients with HM and pneumonia when requiring invasive MV. Throughout the study period, hematological patients haven been initially treated at the department of hematology, including an intermediate care (IMC) ward with 16 treatment units not equipped for invasive MV. If developing ARF, hematological patients have been transferred to the ICU of the department of internal medicine and infectious diseases for more invasive treatment.

We retrospectively enrolled all consecutive adult (≥ 18 years) patients with HM requiring invasive MV due to pneumonia over a period of 12 years (08/2004-07/2016). Information that could identify individual patients were made anonymous after data collection. Because of the anonymous and retrospective analysis the need for participant consent was waved by the ethics committee. `*Pneumonia´* was defined as a clinical diagnosis in combination with pneumonia-suspicious findings on CT scan. Furthermore, we aimed for microbiological proof of pathogens in LRT fluids (endotracheal aspirate, bronchial fluids, or BAL fluids). Patients discharged alive within 24 h after admission were excluded from the analysis because their admission usually occurred only for invasive diagnostics or procedures (e.g. bronchoscopy). Re-admission to the ICU within 10 days after discharge occurred in four cases and was not considered a new case. Later re-admission occurred in three further patients (all after more than one year); these were thus considered new cases.

In case of ARF due to pneumonia with need for intubation and invasive MV standard of care included amongst others invasive microbiological diagnostics (bronchial or BAL fluid, blood cultures), blood parameter analyses and chest CT scans. LRT fluids were tested for: *Aspergillus spp.*, *Pneumocystis jirovecii* (*P. jirovecii*) and bacterial species detected in microbiological culture as well as atypical bacteria detected by polymerase chain reaction (PCR). Seasonal testing for CRV was performed for *Influenza virus*, *Respiratory syncytial virus (RSV)*, *Parainfluenza virus* and *Metapneumovirus* and positive detection of these was considered as viral pneumonia. Additionally, we tested for reactivation of HHV (*Herpes simplex virus 1 (HSV-1)*, *Epstein-Barr-virus (EBV)*, Cytomegalovirus (CMV)). Viral load was assessed by quantitative polymerase chain reaction (PCR), performed on lower respiratory tract specimens (bronchoalveolar lavage or endotracheal aspirate), and results given in copies/ml. PCR was considered as positive with a threshold of > 1.000 copies/ml. With regard to longitudinal monitoring of lung viral load in patients with persistent need for invasive respiratory support, lower respiratory tract fluids were re-tested for viral load at regular intervals at the treating physician’s discretion. Treatment failure for these patients was defined as lack of clinical improvement in combination with failure to achieve at least a significant reduction (> 1 log) in viral load. In individual cases additional testing for serological parameters such as *Aspergillus* antigen (Galactomannan) in serum was performed. Invasive pulmonary fungal disease was considered as positive if it met the European Organization for Research and Treatment of Cancer (EORTC) revised criteria of 2008 for a *probable* or *proven invasive fungal disease* [10]. According to these criteria, the following applied to all of our patients with *probable invasive fungal disease*:

- all our patients had a host factor due to their underlying disease and therapy.
- the clinical criterion was fulfilled through mold-suspicious CT scans.
- the mycological criterion was fulfilled through the cultural detection of Aspergillus in LRT fluids or the detection of Galactomannan in LRT fluids and serum.

In four cases, cultural detection from sterile samples was additionally provided, thus fulfilling the criteria for proven invasive fungal disease.

If more than one pathogenic germ was found, we categorized in primary or secondary/super-infection. CRV were consistently seen as causative for primary infection. If no virus was detectable, the germ detected at the earliest timepoint was considered as causative for primary infection. If detected in LRT fluids, the following germs were considered non-pathogenic commensals (colonization) in accordance with resent guidelines: yeasts, non-hemolytic streptococci, viridans streptococci, coagulase-negative staphylococci and Staphylococcus hemolyticus [22]. If detected, HHV were not rated as pneumonia causing pathogen but as reactivated (except of cases with CT-findings highly suspicious for *CMV* and *HSV-1* pneumonia in combination with very high counts of virus DNA (> 1.000.000 copies/ml) in respiratory tract specimens).

Pneumonia-suspicious CT findings taken up to three days after ICU admission were assessed for diagnosis.

Patients with a total leucocyte count < 1000/µl were categorized as neutropenic.

### Statistical analysis

We analyzed epidemiology of germs and microbiological parameters. At time of discharge from ICU and 1 year after admission we built two groups (survivors and non-survivors) and tested for association of microbiological parameters and mortality with Pearson´s Chi-Quadrat test. If less than 5 patients built a group, we used Fishers Exact Test. After univariate analysis we tested for collinearity and multivariately analyzed the risk factors for mortality by using logistic regression. Findings were regarded as statistically significant, if p-value was < 0.05. Kaplan-Meier-curves were calculated for survival analyses by using LogRank-test.

Software used for data collection and analysis were: *IS-H med.* (SAP SE, Walldorf, Germany), *Lauris Order Communication System* (Nexus AG, Donaueschingen, Germany), *PACS Universität Heidelberg* (GE Medical, Chicago, Illinois, USA), Microsoft *Access* (Microsoft Corporation, Redmond, Washington, USA), Microsoft *Excel* (Microsoft Corporation, Redmond, Washington, USA), IBM *SPSS Statistics 24* (IBM Corporation, Armonk, New York, USA) and *EndNote* (Clarivate Analytics, London, England).

This study was approved by the local ethics committee of the University of Heidelberg, Germany (authorization number: S-457/2015).

## Results

246 patients with HM requiring invasive MV due to pneumonia were included in this analysis. Four patients were lost to follow-up and not included in 1-year survival analysis. ICU and 1-year survival were 37.0% (91/246) and 19.0% (46/242), respectively. 97 (39.4%) patients were female. At ICU admission median age was 58 years and 117 (47.6%) patients were post-HSCT. Baseline characteristics of the study cohort are displayed in Table 1.

**Fig. 1:**
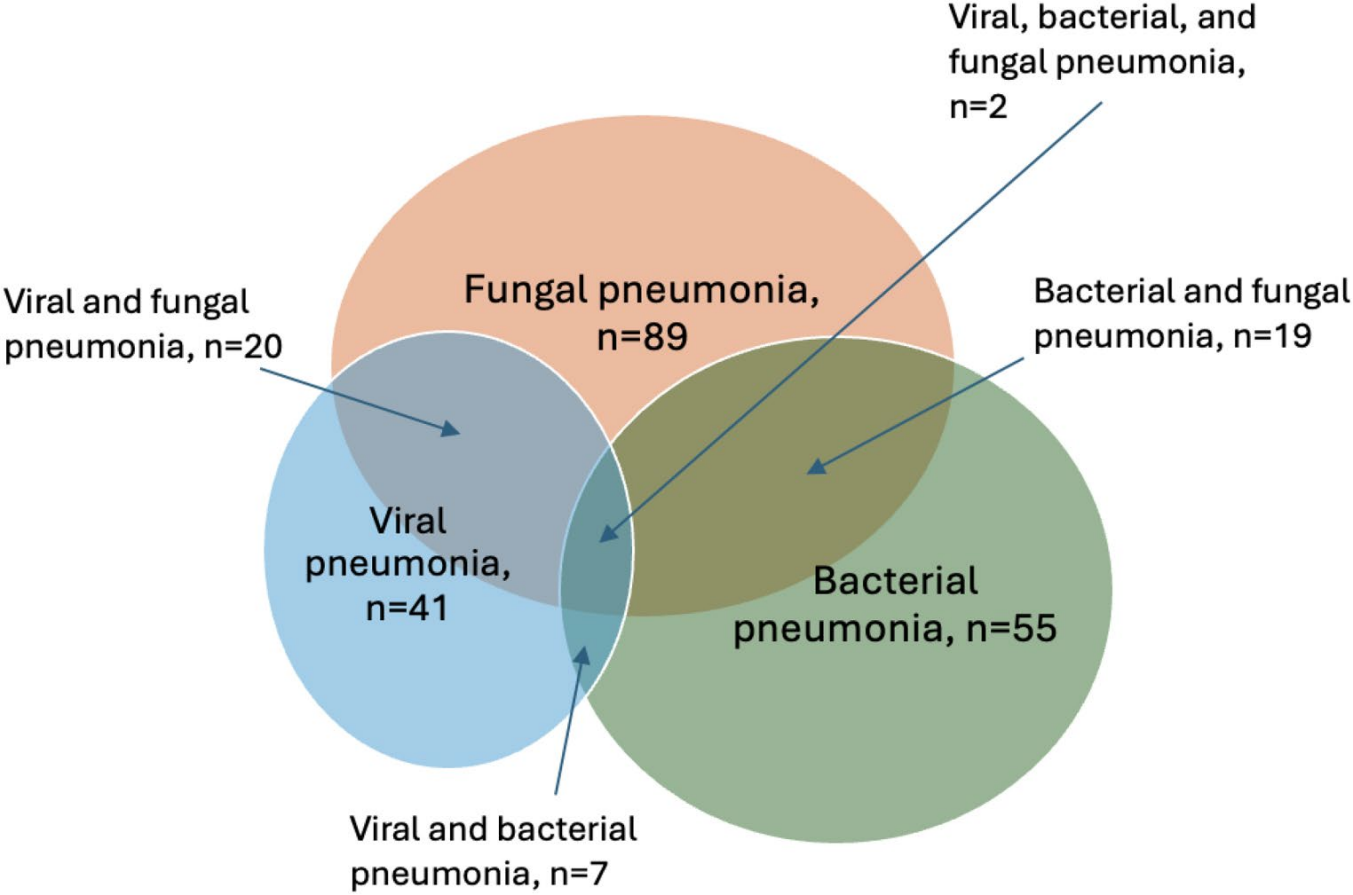
Figure 1 displays the number of all detected pathogenic organisms in a Venn diagram. The overlapping circles indicate which groups of pathogens were frequently detected together in co-infections

For all included patients at least one LRT specimen was available for analysis. In 103 (41.9%) patients, the diagnosis of pneumonia was based on pneumonia-suspicious findings in CT scans combined with clinical symptoms, without microbiological proof. In addition to clinical and radiological findings, one or more pathogenic agents were detectable in 143 (58.1%) patients. Fungal, bacterial and viral infections were detected in 89 (36.2%), 55 (22.4%) and 41 (16.7%) patients, respectively (see Fig. 1). 51 (20.7%) patients had multimicrobial pulmonary infections. Reactivation of HHV were detected in 85 (34.6%) patients (see Table 2).

**Table 1:** Baseline characteristics and ICU and 1-year survival of the cohort and subgroups

	Number of patients [n]	% of total cohort	ICU survival [n] (%)	1-year survival [n] (%)
Total cohort	246		91 (37.0%)	46 (19.0%)
Hematological diseases				
AML	78	31.7%	27 (34.6%)	12 (15.8%)
NHL	66	26.8%	22 (33.3%)	9 (13.8%)
Multiple myeloma	40	16.2%	22 (55%)	14 (35.9%)
MDS	18	7.3%	4 (22.0%)	0 (0%)
ALL	17	6.9%	7 (41.2%)	3 (17.6%)
Hodgkin´s lymphoma	8	3.3%	3 (37.5%)	3 (37.5%)
AL amyloidosis	4	1.6%	2 (50%)	2 (50%)
CML	3	1.2%	1 (33.3%)	0 (0%)
Others	12	4.9%	3 (25%)	3 (25%)
Patients post-HSCT at ICU admission	117	47.6%	33 (28.2%)	17 (14.7%)
post-allogeneic HSCT	86	35.0%	19 (22.1%)	7 (8.1%)
post-autologous HSCT	31	12.6%	14 (45.2%)	10 (33.3%)
gvhd	52	21.1%	12 (23.1%)	4 (7.7%)
Sex: female	97	39.4%	35 (36.1%)	15 (15.6%)
Sex: male	149	60.6%	56 (37.6%)	31 (21.2%)
Length of stay at ICU [days]	median: 8 (range 1-160)			
Duration of invasive mechanical ventilation [days]	median: 6 (range 1-116)			

1-year survival: 4x lost to follow up

*AL amyloidosis*: *Amyloid light-chain amyloidosis*, *ALL*: *Acute lymphoblastic leukemia*, *AML*: *Acute myeloid leukemia*, *CML*: *chronic myeloid leukemia*, gvhd: graft versus host disease, HSCT: hematopoietic stem cell transplantation, ICU: Intensive Care Unit, *MDS*: *myelodysplastic syndrome*, n: number of patients, *NHL*: *Non-Hodgkin´s-lymphoma*

### Fungal pneumonia

In 89 (36.2%) patients with pneumonia, a pathogenic fungal germ in respiratory specimens could be diagnosed (see Table 2). According to the EORTC criteria, four cases were classified as *proven* and 85 as *probable invasive fungal disease* [10]. ICU and 1-year survival of patients with fungal pulmonary infections were 37.1% (33/89) and 19.8% (17/86), respectively. *Aspergillus spp.* were most frequently identified (*n* = 68) by positive Galactomannan (GM) and cultural proof in LRT fluids. Of 68 patients with *pulmonary Aspergillus disease*, 37 (54.4%) were tested positive for GM in serum as well. ICU survival was very low in these patients with positive GM in serum with only 21.6% (8/37).

### Bacterial pneumonia

In 55 (22.4%) patients pathogenic bacteria were detected in LRT specimens (see Table 2). ICU survival of patients with bacterial pneumonia was 45.5% (25/55), 1-year survival 14.8% (8/54). In 20 (36.5%) patients with bacterial pneumonia, the pneumonia-causing bacterium was also detectable in blood culture. *Pseudomonas spp.* was identified most frequently (*n* = 24), followed by *Klebsiella pneumoniae* (*n* = 7). Bacteria with multi-drug resistance (MDR) (*Methicillin-resistant Staphylococcus aureus*, extensively drug-resistant (XDR) *gram-negative bacteria*) were found in 17 (6.9%) patients.

### Viral pneumonia

Respiratory viruses were identified in 41 (16.7%) patients (see Table 2). Detection of respiratory viruses was associated with an ICU survival of 22.0% (9/41) and 1-year survival of 12.2% (5/41). *RSV* (*n* = 16) and *Influenza* (*n* = 14) were the most frequently identified respiratory viruses.

In five cases HHV (3x *CMV*, 2x *HSV-1*) were found as primarily causative for pneumonia. In these five cases CT-findings were highly suspicious for *CMV* and *HSV-1* pneumonia in combination with very high counts of virus DNA (> 1 000 000 copies/ml) in LRT specimens.

### Detection of human herpesviruses reactivation

Concomitant reactivation of HHV was detected in LRT fluids of 85 (34.6%) patients. Associated ICU and 1-year survival was 38.8% (33/85) and 23.2% (19/82), respectively. *CMV*, *EBV*, and *HSV-*1 were detectable in 28 (11.4%), 16 (6.5%), and 50 (20.3%) patients, respectively. In 18 of the *CMV*-positive patients *CMV* was detectable at intubation timepoint while in 10 patients *CMV* reactivation only occurred at a later stage. Associated survival data from patients with HHV differ much and are shown in Table 2.

**Table 2:** Pneumonia causing pathogens in lower respiratory tract fluids

	Number of patients [n]	% of total cohort	ICU survival [n] (%)	1-year survival [n] (%)
Total cohort	246		91 (37.0%)	46 (19.0%)
Undefined pneumonia	103	41.9%	39 (37.9%)	21 (20.6%)
Pneumonia causing germs identified	143	58.1%	52 (36.4%)	25 (17.9%)
Fungal pneumonia	89	36.2%	33 (37.1%)	17 (19.8%)
*Aspergillus spp.* pneumonia*	68	27.6%	23 (33.8%)	10 (15.4%)
Asper. cultural proof in LRT	35	14.2%	11 (31.4%)	5 (14.7%)
Galactomannan in LRT	25	10.2%	9 (36%)	4 (16.7%)
Galactomannan in serum	37	15.0%	8 (21.6%)	3 (8.3%)
*Pneumocystis jirovecii*	19	7.7%	11 (57.9%)	7 (36.8%)
*Mucorales*	6			
Other fungi	6			
Bacterial pneumonia	55	22.4%	25 (45.5%)	8 (14.8%)
Non-MDR bacteria	38	15.5%	19 (50%)	7 (18.9%)
MDR bacteria	17	6.9%	6 (35.3%)	1 (5.9%)
Blood-culture positive	20	8.1%	8 (36.5%)	3 (15%)
Blood-culture negative	35	14.3%	17 (48.6%)	5 (14.7%)
Viral pneumonia	41	16.7%	9 (22.0%)	5 (12.2%)
*RSV*	16	6.5%	3 (18.8%)	1 (6.3%)
*Influenza*	14	5.7%	3 (21.4%)	1 (7.1%)
*Parainfluenza*	7			
*Other viruses*	6			
Reactivation of human herpesviruses	85	34.6%	33 (38.8%)	19 (23.2%)
*HSV-1* reactivation	50	20.3%	25 (50%)	14 (28.8%)
*EBV* reactivation	16	6.5%	5 (31.3%)	1 (6.7%)
*CMV* reactivation	28	11.4%	8 (28.6%)	4 (14.8%)
*CMV* reactivated at ITN	18	7.3%	2 (11.1%)	0 (0%)
Multimicrobial infections**	51	20.7%	18 (35.3%)	7 (14%)
Viral pneumonia + *Aspergillus spp.* superinfections	19	7.7%	4 (21.1%)	2 (10.5%)
*RSV + Aspergillus spp.*	8			
*Influenza + Aspergillus spp.*	6			
Other viruses + *Asp. spp*.	5			

1-year survival: 4x lost to follow up

*Asp.*: *Aspergillus*, *Aspergillus spp*.: *Aspergillus* species pluralis, *EBV*: *Epstein Barr virus*, *CMV*: *Cytomegalovirus*, HHV: *human herpesviruses, HSV-1*: *herpes simplex virus* 1, ICU: Intensive Care Unit, ITN: intubation, LRT: lower respiratory tract, MDR: multi-drug resistance, n: number of patients, *RSV*: *Respiratory syncytial virus*

*Aspergillus spp. pneumonia was rated positive in case of positive mycological criterion (cultural proof in LRT fluids and/or Galactomannan proof in LRT fluids and Galactomannan proof in serum) and positive clinical criterion (mold-suspicious computed tomography scans) according to the revised EORTC criteria of *probable invasive fungal disease* [10].

**In terms of microbiological parameters, 51 patients were counted multiple times in the pathogen groups ‘fungal’, ‘bacterial’, and ‘viral’, as well as in ‘multimicrobial infections’, due to the presence of polymicrobial infections.

### Multimicrobial pulmonary infections

In 51 (20.7%) patients more than one pathogenic germ was detected. In Table 2, patients with multimicrobial pneumonia are listed separately for each bacterial, fungal, and viral pathogen. Therefore, in Table 2, the total number of pathogens is higher than the total number of patients with detectable pathogens. ICU survival of these patients was 35.3% (18/51), 1-year survival was 14% (7/50). Secondary infections were fungal, bacterial and viral in 34, 24 and 3 patients, respectively.

In 19 patients with multimicrobial infections primary infections were caused by respiratory viruses with later development of *Aspergillus spp.* superinfections. ICU survival of these patients was 21.1% (4/19) and 1-year survival only 10.5% (2/19) (see Table 2).

### Subgroup analysis of patients with HSCT

117 (47.6%) patients received HSCT before ICU admission. Pathogenic germs were identified in 65.8% (77/117) of patients (see Table 3). Viral infections were significantly more frequent in post-transplant patients than in patients without HSCT (26% vs. 9%, *p* < 0.001). Multiple germs causing pneumonia were found in 31 (26.5%) post-transplant patients. Thus, multimicrobial infections were significantly associated with post-HSCT status compared to non-transplant patients (27% vs. 16%, *p* = 0.042). Furthermore, post-HSCT status was significantly more frequent in patients that died in the ICU than in ICU survivors (55% vs. 36%, *p* = 0.004).

**Table 3:** Comparison of total cohort with subgroup of patients with HSCT

	Patients with HSCT [n] (%)	Total cohort [n] (%)	Significance (*p*-value)	
Number of patients	117 (100%)	246 (100%)		
ICU survival	33 (28.2%)	91 (37.0%)	0.004*	
1-year survival	17 (14.7%)	46 (19.0%)	-	
Undefined Pneumonia	40 (34.2%)	103 (41.9%)	-	
Pneumonia causing germs identified	77 (65.8%)	143 (58.1%)	0.025*	
Fungal pneumonia	47 (40.2%)	89 (36.2%)	-	
Bacterial pneumonia	25 (21.4%)	55 (22.4%)	-	
Viral pneumonia	30 (25.6%)	41 (16.7%)	< 0.001*	
Multimicrobial pneumonia	31 (26.5%)	51 (20.7%)	0.042*	
Reactivation of human herpesviruses	46 (39.3%)	85 (34.6%)	-	

*level of significance: <0.05

Regarding microbiological parameters 51 patients were multiply counted because of multimicrobial infections

1-year survival: 4x lost to follow up

*HSCT* hematopoietic stem cell transplantation, *ICU survival* Intensive Care Unit survival, *n* number of patients

### Microbiological risk factors for mortality

In univariate analysis ICU Non-survivors had significantly more often a viral pneumonia (21% vs. 10%, *p* = 0.029), a *probable invasive Aspergillus disease* in combination with positive serum-GM (19% vs. 9%, *p* = 0.036) and a *CMV* reactivation at intubation timepoint (10% vs. 2%, 0.013). Patients not surviving one year had significantly more often a *CMV* reactivation at intubation in univariate analysis (9% vs. 0%, *p* = 0.019) but showed no significant differences with respect to viral pneumonia and *probable invasive Aspergillus disease* frequencies (see Table 4).

**Table 4:** Microbiological risk factors for mortality (univariate analysis)

Microbiological risk factor	ICU Non-Survivor	ICU Survivor	*p*-value	1-year Non-Survivor	1-year Survivor	*p*-value
Viral pneumonia	20.6%	9.9%	0.029*	18.4%	10.9%	0.222
*PIAD* + GM in serum	18.7%	8.8%	0.036*	16.8%	6.5%	0.054
*CMV* reactivation at intubation	10.3%	2.2%	0.013*	9.2%	0.0%	0.019*

*level of significance: <0.05

*CMV* Cytomegalovirus, *GM* Galactomannan, *ICU survival* Intensive Care Unit survival, *n* number of patients, *PIAD* probable invasive Aspergillus disease

**Fig. 2:**
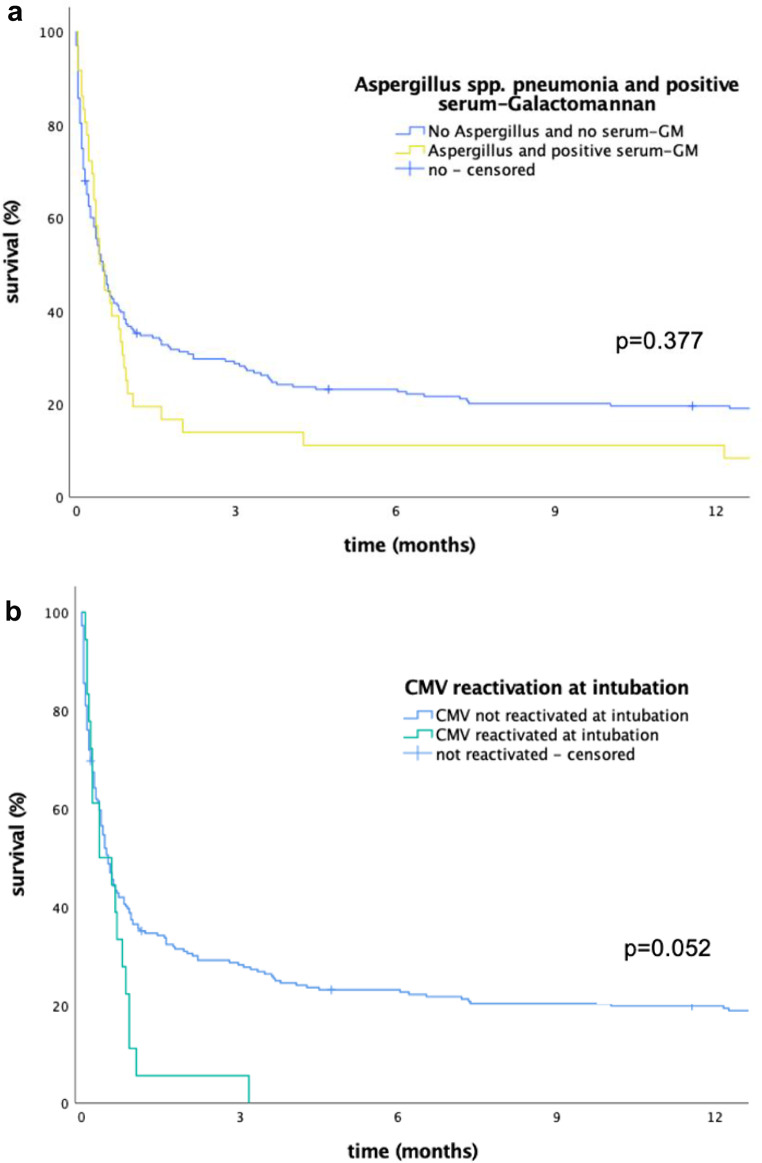
Figure 2a displays the survival function of patients with and without Aspergillus spp. pneumonia and positive serum-Galactomannan (LogRank test). Figure 2b displays the survival function of patients with and without *CMV* reactivation at intubation timepoint (LogRank test)

In multivariate analysis the following microbiological risk factors for ICU mortality were found: *Probable invasive Aspergillus disease* in combination with positive testing for GM in serum (*p* = 0.021, odds ratio 3.1 (1.2-8.0)) and *CMV* reactivation at intubation timepoint (*p* = 0.043, odds ratio 5.3 (1.1–26.8)) (see Table 5). Long-term survival was not significantly associated with these microbiological risk factors in multivariate analysis (see Fig. 2a and b).

**Table 5:** Microbiological risk factors for ICU mortality (multivariate analysis)

	Odds ratio	
Microbiological risk factor	(95% Confidence interval)	*p*-value
Viral pneumonia	2.020 (0.801–5.089)	0.136
*PIAD* + Galactomannan in serum	3.075 (1.184–7.986)	0.021*
*CMV* reactivation at intubation	5.321 (1.057–26.796)	0.043*

*level of significance: <0.05

*CMV* Cytomegalovirus, *ICU mortality* Intensive Care Unit mortality, *PIAD* probable invasive Aspergillus disease

## Discussion

In this study high ICU mortality of 63% in patients with HM requiring invasive MV due to pneumonia was found. Microbiological parameters significantly associated with death at ICU were *probable invasive Aspergillus disease* with positive serum-GM (both as primary infection and as superinfection on CRV) and *CMV* reactivation at intubation timepoint with associated ICU mortality of 78% and 89%, respectively. 1-year survival of the cohort was only 19%. In multivariate analysis, there were no microbiological risk factors significantly associated with 1-year survival.

In line with our findings of an ICU survival rate of 37%, other studies have also observed high mortality rates among patients with hematologic malignancies admitted to the ICU due to respiratory infections, with hospital survival rates ranging from only 30–40% [23–25]. Hence, our study adds further proof of high mortality of infectious complications leading to need for critical care support in patients with HM. Contrary to short-term survival, we did not find a significant association between microbiological factors and 1-year survival. Similarly, other studies have shown that the long-term survival of patients with HM is not compromised by an acute, ICU-requiring illness or complication, such as respiratory infections, provided that it is survived [26].

Knowing causative germs for pneumonia helps to treat patients more efficiently and can improve the outcome [27]. Thus, invasive diagnostic testing (e.g. BAL) is important for identifying causative germs as early as possible and providing adequate anti-infective therapy. However, in this study LRT fluids were analyzed extensively in all patients but pneumonia causing pathogens were identified only in about half of patients. Other studies had similar findings [12, 20]. In these cases, empirical therapy should be continued or (even without germ detection) adapted to radiological findings, the local spectrum of germs, and local drug resistances.

In hematological patients, fungal pneumonia, particularly due to *Aspergillus spp.*, is associated with high mortality [12, 28]. Our present study with ICU mortality of 66% in patients with pulmonary Aspergillosis underlines that. In combination with proof of GM in serum, *Aspergillus spp.* pneumonia was a significant risk factor for ICU mortality. In line with our findings, Ledoux et al. recently verified the diagnostic value of GM testing in BAL and blood samples [28]. In addition, our findings indicate that proof of GM in serum in patients diagnosed with pulmonary aspergillosis is not only a diagnostic but also an outcome-relevant parameter. Nevertheless, the significance of GM testing is discussed controversially and positive testing is not considered as *proven invasive aspergillosis* in the latest EORTC/MSGERC definitions of invasive fungal disease [29]. Prospective studies will help to understand the diagnostic and prognostic value of GM testing.

Multimicrobial pulmonary infections are often seen in immunocompromised patients and complicate the anti-infective therapy. In this study multimicrobial pneumonia was detected in 20% of patients. In particular, patients suffering from pneumonia with CRV and consecutive *Aspergillus spp.* superinfections showed high ICU mortality. Prevalence and mortality of multimicrobial pneumonia were similar in other studies [20, 30]. They also found pneumonia with CRV frequently associated with *Aspergillus spp.* superinfections with high mortality [20, 31, 32], a lesson learned also during the recent COVID-19 pandemic [33]. Thus, physicians should monitor for fungal superinfections in patients with viral pneumonia and ARF.

Prior to the *SARS-CoV-2* pandemic, *Influenza* was primarily seen as the CRV predisposing for *Aspergillus spp.* superinfections [34]. However, in this pre-COVID-19 pandemic study, the frequently detected constellation was *RSV* pneumonia with subsequent *Aspergillus spp.* superinfections. Similar to our findings, Magira et al. could show high mortality in hematological patients having this specific infectious constellation [35]. Hence, in addition to Influenza and SARS-CoV-2, RSV appears to be another respiratory virus causing high morbidity and mortality, especially when combined with Aspergillus spp. superinfections. Prospective studies with more patients will help to find out if this infectious constellation (CRV and Aspergillus spp.) is a significant risk factor.

Concomitant reactivation of HHV in critically ill patients is a frequent finding [36]. Many efforts have been undertaken to evaluate the risk especially for *CMV* reactivations in patients with HM [14]. We found *CMV* reactivations in the lung associated with high mortality. When *CMV* reactivation was detected early (at intubation timepoint), it was a significant risk factor for ICU mortality. Pinana et al. could also show that *CMV* DNAemia in context with ARF represents a risk factor for poor survival [30] while other studies showed inconclusive findings concerning the association between *CMV* reactivation and mortality [14]. However, our findings indicate that not just the proof of *CMV* but the time context helps to interpret the mortality-risk associated with *CMV* reactivation.

Due to the retrospective study design, there are several limitations. There was no standardized protocol for collecting microbiological and radiological data. Furthermore, our findings were not externally validated. Although our strict definition of pneumonia (radiologically proven) makes it more comparable to other studies, it bears the risk of underdiagnosis, as pulmonary infiltrates are frequently challenging to recognize and interpret in hematological patients. Due to altered immune defense and frequent past antimicrobial therapies, hematological patients may experience colonization with facultatively pathogenic organisms. Therefore, the possibility of misinterpreting microbiological findings cannot be completely ruled out. ICU survival as a short-term parameter for mortality is not capable of fully attributing cause and effect. Other causes of mortality, such as infections outside the lungs or comorbidities, must be considered as confounders. However, the impact of the COVID-19 pandemic on the intensive care management of hematologic patients, along with the associated complications and mortality, has been demonstrated in other studies and should be considered when interpreting the results of our study [37].

## Conclusion

In this study high mortality in patients with HM requiring invasive mechanical ventilation due to pneumonia was found. In particular, patients suffering from multimicrobial pneumonia with CRV and consecutive *Aspergillus spp.* superinfections showed high ICU and 1-year mortality. However, pulmonary Aspergillosis with positive GM in serum and *CMV* reactivation in context with ARF could be identified as microbiological parameters significantly associated with ICU mortality.

## Data Availability

Data not publicly available.
